# Person–Environment Fit in Urban Neighbourhoods in Slovenia: Challenges and Coping Strategies

**DOI:** 10.3390/ijerph20065139

**Published:** 2023-03-14

**Authors:** Maša Filipovič Hrast, Richard Sendi, Boštjan Kerbler

**Affiliations:** 1 Faculty of Social Sciences, University of Ljubljana, 1000 Ljubljana, Slovenia; 2 Urban Planning Institute of the Republic of Slovenia, 1000 Ljubljana, Slovenia

**Keywords:** person–environment fit, urban neighbourhoods, ageing in place, coping strategies, central and eastern Europe

## Abstract

A poor person–environment fit may bring various negative effects to older people’s independence and physical and psychological well-being. The presented study is especially valuable as it explores the challenges of living in cities in a country located in central and eastern Europe; namely, a less researched area when it comes to the quality of life of older people dwelling in an urban environment. The research questions that were explored are (1) what environmental pressures have people identified in the urban environment in Slovenia; and (2) what strategies have they used to deal with them? The study is based on 22 interviews with older people and three focus groups, that were then analysed using a thematic analysis approach. The study results identify a number of environmental pressures, which were divided into structural housing pressures, structural neighbourhood pressures, and formal and informal pressures. The analysis shows important behavioural responses, such as strategies of using formal and informal help, moving away from environmental pressures, mobility, actively involved in changing the environment, as well as attitudinal adaptation strategies, such as acceptance, resilience, using distraction, modesty and planning for the future. We further emphasize how these coping strategies are linked to individual and community capabilities, which function as a conversion factor.

## 1. Introduction

Population ageing is one of the most pronounced demographic processes in modern society. Yet, the world is simultaneously witnessing the rapid growth of cities and urban populations. Urban development and population ageing should be two closely linked and interconnected processes [1]. Rapid urbanisation means that in the future, cities will provide a home for a large share of the older population [2]. Only well-planned cities can ensure the facilities needed to improve the quality of life of older residents. Creating an appropriate urban and community environment for older residents is thus essential if cities are to be liveable [3]. The Brasilia Declaration on ageing, adopted by the World Health Organization (WHO) in 1996, states that healthy, older persons are a resource for their families, their communities and the economy” [4]. The more active such persons are, the more they can contribute to society [5]. This was also one of the three priorities of the sustainable human development plan presented at the second international conference on ageing in 2002 called Building a Society for All Ages, and organised by the UN in Madrid. Cities adapted to the needs of an ageing society, also known as “age-friendly cities”, are hence a necessary and logical response to promoting the well-being and quality of life of the older people in urban environments, where older residents can function as a social resource that adds to the city’s success. Age-friendly cities are defined by how well various areas contribute to the quality of life of older people. These areas range from transportation adapted for older people to the accessibility of housing and community services. Outdoor spaces are also important, especially in terms of safety and accessibility, opportunities for older people to actively participate in society and access to communication and information [6]. At that, urban neighbourhoods face several challenges in their efforts to develop a supportive environment for older people, such as growing economic inequalities, a lack of social support networks, and the impact of both welfare changes and economic austerity on urban neighbourhoods [7]. Deprived urban areas are associated with higher levels of social exclusion in old age [8]. The goal of ageing in place insufficiently recognises the challenges older people encounter in the areas where they live that may be poorly adapted to older people’s changing needs, despite the positive effects that ageing in place can have on feelings of security, independence and autonomy [9,10,11]. This makes it important to observe how older people and the environment fit, what particular pressures do people encounter in their environment and how they respond to them.

Environmental gerontology seeks, among others, to identify relevant dimensions of the environment and the mechanisms by which the environment shapes individual ageing, while also encompassing a person’s response, experience and adaptations [12]. The relationship between home and its surroundings and an older person is emphasised in the ecological theory of ageing [13]. It defines the process as constantly adapting to changes in the environment and the altering of one’s own behaviours, highlighting five key components: (1) individual ability; (2) environmental pressure; (3) adaptive behaviours; (4) affective responses and (5) adaptation level. Phillips et al. [14] distinguish the structural domain of the person–environment (P–E) fit model, i.e., housing, neighbourhood characteristics, the informal domain, i.e., social support, relationship with family, friends, and neighbours, as well as the formal domain, i.e., various services. The strength of the ecological theory of ageing is in recognition of the transactional nature of an individual and their contextual resources [15]. High-quality ageing within a neighbourhood requires a person–environment fit or interaction between an individual’s abilities and the environment’s social and physical characteristics that determine how successfully an older person will be able to live in a community. 

However, person–environment fit is not a static process whereby the person or environment can be observed as a constant and both must be observed as (potentially) changing simultaneously. Furthermore, P–E fit must be observed in specific national, cultural and structural (housing and other characteristics) contexts because they affect how this fit is established. Phillips et al. [14] revealed how cultural context and values can affect how P–E fit is evaluated by older people, indicating the relevance of, for example, family values as a moderating influence in P–E fit. A specific value of the article lies in investigating the challenges of living in cities in a country located in central and eastern Europe; namely, a less researched area when it comes to older people’s quality of life while dwelling in an urban area. Kovacs [16] noted that research into mobility and the housing situation in post-socialist cities is rare, with under-researched issues relating to certain population groups, such as older people. Historical factors influencing the housing market (building process in urban areas and mass privatisation) mean that older people in post-socialist cities tend to concentrate in the older, lower quality segment of the housing stock, which makes the need for renovation and potential problems with the dwellings more likely [16]. 

Slovenia has the special characteristic of being a “super” homeownership society, with very low mobility [17,18,19]. Extremely radical privatisation of the former state-owned rental housing followed the country’s independence in 1991. According to Mandič [19], 96% of Slovenians over 65 years of age own their apartment or house. With age, and especially above the age of 80, the share of homeowners even rises. Following the Second World War and especially in the 1960s and 1970s, people moved away from the rural, less developed and remote areas to towns and other employment centres, causing accelerated urbanisation [20]. In 2014, when the national statistical office last published data for urban and non-urban settlements, almost 53% of people aged 65 or over lived in an urban settlement, while slightly more than 47% lived in a non-urban settlement [21]. In 2015, a large-scale survey was conducted in Slovenia regarding the housing of older people [22]. The survey showed that almost 60% of people aged 65 and over in urban areas were living in multi-apartment buildings, and up to 95.3% of them were not living on the ground floor. It is particularly worrying that 60% of these people were living in a building without an elevator [20]. In addition, a survey on the social exclusion of older people [23] showed that, compared to other EU countries, Slovenia has a significantly higher share of older people who are spatially excluded—that is, they do not have access to basic services in their local environments, which is a key obstacle to high-quality ageing in these environments and increasing the dependence of older people. In the 2015 survey [22], nearly half of older people (49%) reported their spatial exclusion. 

These conditions and challenges mean that it is vital to understand the specifics of person–environment fit in these circumstances. The focus of this article is on exploring the challenges within the urban environment in Slovenia based on the person–environment fit theoretical approach. The aim of the paper is to present the findings of a case-study evaluation of the person–environment fit which was conducted in Slovenia. As one of the post-socialist countries and given the similar ideologies and principles that were applied to the broader region in the planning and development of urban residential neighbourhoods [24,25,26,27,28,29], the study conducted in Slovenia has the potential to introduce new knowledge and a better understanding of the complex contextual influences on person–environment fit. The study was based on two key research questions:1) what environmental pressures did people identify in the urban environment in Slovenia and 2) what strategies did they use to deal with them? 

## 2. Methods

### 2.1. Study Design

The data for this study were obtained by conducting three focus groups (FGs) and 22 interviews. We decided on both qualitative methods of data collection because focus groups enable the exchange of opinions and discussion, while personal interviews enable more in-depth questioning [30,31,32], combining individual interviews and focus groups to enhance data richness. Participants were older people aged 65 and over living in different urban environments, hence in larger and smaller cities, in apartments or houses. Half of the interviews were conducted in larger cities and half were conducted in smaller ones. Two focus groups were carried out in a larger city (Ljubljana), one with older people living in apartments and the other with those living in houses. The third focus group was conducted in a smaller town (Žalec). Individual interviews lasted between 1 and 1.5 h and the FGs ran for approximately 1.5 h.

The questions in the focus groups and individual interviews were similarly directed toward observing problems in their immediate environment and home. Those for the focus groups were fewer and more condensed, with more focus on the discussion of possible policy solutions. In the interviews, discussions were more detailed and included more individualised strategies. 

In the interviews, the respondents described their life in their current home, satisfaction with their housing and environment, their typical day, the problems they were facing in their surroundings and how they were dealing with them, the support they were receiving, and thoughts and wishes for the future. The FG participants similarly discussed their satisfaction with their living environment and the problems they were encountering, how they were dealing with them and the support they were receiving. They discussed the future and their desires, and a selection of good practice examples, which however are excluded from this analysis.

### 2.2. Recruitment and Participants

Participants for the focus groups were recruited with the help of non-governmental organisations. In the larger city (Ljubljana), we were able to secure the help of the Third Age University, and in the small town (Žalec), we obtained the help of the Slovenian Philanthropy. The focus groups were moderated by a member of the project team who had the relevant appropriate competences and experience. In addition to the moderator, an additional researcher also participated in supporting the process and ensuring research quality. Participants for interviews were obtained with the help of students from the Faculty of Social Work, University of Ljubljana. Students enrolled in the course Social Work With Older People had a special seminar where they acquired the necessary knowledge for conducting interviews. These students were also generally trained in conducting research with vulnerable population groups and in the ethics of social work through their studies. 

### 2.3. Data Collection and Analysis

The FGs were conducted in April 2019. Almost all participants of the focus groups were women, except in the Ljubljana FG (living in houses) where there was one male participant. All participants lived independently in their home environments. The interviews were conducted from December 2018 to January 2019. The average age of the interviewees was 77.5, at 13 were women and nine were men. Of these, four had primary school education, 12 had vocational or a similar level of education and six had higher education. The interviews and focus groups were recorded and transcribed. The analysis followed the approach of a thematic analysis, which enables recognition, analysis and interpretation patterns of meanings or topics in qualitative data [33,34]. In accordance with the process of thematic analysis, as described by Braun and Clarke [33], and with the help of the NVivo computer program, we first encoded the data obtained and based on this, we identified topics for the challenges people face in the urban environment and strategies used to cope with them. These were, in the following step, merged into specific categories. For greater validity of the analysis, the data was analysed by two researchers, thus ensuring the unification of the code list. The independent analysis performed by two researchers reduces the possibility of bias and at the same time increases the interpretative basis of the research [35]. 

## 3. Results

### 3.1. Environmental Pressures Identified in Urban Areas in Slovenia

The results summarise the analysis of the qualitative data along three lines. First, they present the environmental pressures that were identified by the thematic analysis and later grouped into themes and categories (see Table 1). We divided these pressures into the following categories: structural, i.e., housing, neighbourhood characteristics, the informal domain, i.e., social support, relationship with family, friends and neighbours, and the formal domain, i.e., various services.

#### 3.1.1. Structural Environmental Pressures—Housing

Among the structural environmental pressures associated with housing, respondents emphasised the problems of accessibility, such as the non-existence of ramps, multi-apartment buildings without elevators, too many stairs, etc., or the size of the dwellings, as indicated in the interviews:

“I would reduce it by half, I put the sleeping quarters downstairs, so I don’t have any stairs, there are living rooms up top, there is another apartment up top, so it is too big for two people, I would have a smaller one, it is too much to clean, the heating costs are high for such a house, for two people.” (Interviewee D8)

Furthermore, several people mentioned that there were several problems linked to the upkeep of the dwelling, the age of the dwelling and financial problems related to high levels of poverty among older people [36], which often make the excessively big houses or apartments hard to maintain, leading to dampness, high heating costs, poor sound isolation, and deteriorating appliances and furniture.

#### 3.1.2. Structural Environmental Pressures—Neighbourhood

The respondents raised several issues that primarily relate to the walkability of the neighbourhood and the limitations caused by a lack of benches to sit on, busy roads and sometimes hilly terrain. A built environment featuring poor or absent accessibility for older people is an indication of ageism in the urban environment [37]. As one interviewee noted:

“Older people come here… there is absolutely no way for an older person who has problems with their legs or with walking to sit down decently and rest. I’m sorry, this is completely wrong and whoever planned this...” (Focus group LJ, B6)

Another aspect of the neighbourhood concerned its maintenance or lack thereof, cleanliness, noise (from traffic or loud groups of young people partying) and other similar environmental pressures. In some cases, these aspects were viewed positively while others were critical, e.g., a lack of trash bins, noise.

“Mowing the grass, tidiness, they clean, for example, the park that we have nearby, it is well maintained, so we have absolutely no comments about the tidiness of the surroundings” (Interview B8);

“Noise emissions have increased significantly” (Interviewee B7);

“Traffic has increased, the traffic” (Interviewee B4).

Feelings of safety are an especially important aspect in large dense urban areas and were described by the interviewees as a reduced feeling of safety due to vandalism and theft, or poorly lit walkways. Feeling unsafe was stressful in a few cases:

“Well, I would add that the road, this XXX road, is untidy, and not all the streets have public lighting. I am often afraid to go home from the bus station in the evening because it is an unlit street” (Focus group LJ, B2).

#### 3.1.3. Formal Environmental Pressures—Services

One of the main issues was the accessibility of transportation, which was often vital to access various services. This is therefore closely related to the noted lack of various services in the neighbourhood and the need for public transportation, which was often found insufficient: “I have a very big comment about this. We have the public bus number 8, but it is so rare that I prefer to go to the city by car…” (Focus group LJ, B2). 

Furthermore, it was not only this aspect of the presence/absence of services that was discussed, but interestingly the data also indicated how important the issue is of quality and familiarity with the services. The interviewees showed a preference for smaller shops that would allow for this feeling of familiarity to be established and enable the employees and customers to have superficial knowledge of each other:

“There is a lack of smaller shops. Smaller shops, local ones compared to these large markets, were all closed, Store XX had a large one in area YY, but it was closed (…). Older people feel better in smaller stores where you have contact with the seller and so on.” (Focus group LJ, B1)

Another aspect of quality is the problem of finding good services and preventing bad experiences when using services needed (e.g., small repairs and similar). Older people would appreciate easy access to such information since, despite the digital age we are living in and the access to information regarding various services available on the internet, this seems to pose a specific challenge for older people.

#### 3.1.4. Informal Environmental Pressures

One of the noteworthy informal environmental pressures is the lack of support from the family usually caused by distance from family networks. Another aspect of informal environmental pressure concerned the relationship with the neighbours where the problem of unfriendliness and poor relationships was mentioned. Yet, interestingly, another aspect was the issue of having someone in the neighbourhood with an ‘overview of things’: “I’ve already thought about it, our neighbourhood quarter to function as an umbrella, as regards our environment and life they should have an overview, (…) I wish that they could have a collection of these various craftsmen from various fields, who would be verified” (Focus group LJ, B3).

### 3.2. Responses to Environmental Pressures—Coping Strategies

In this section, we focus on the coping strategies that older people in Slovenia use as a way to respond to the environmental pressures. We summarised this in two main categories: behavioural strategies/responses, and attitudinal strategies/responses (see Table 2).

#### 3.2.1. Behavioural Responses

We identified several strategies within the behavioural responses to environmental pressures. People commonly rely on informal help, mostly from family and neighbours, but also formal services, as well as moving home to reduce environmental pressures and actively changing the environment.

- Strategies using formal help. Use of the available formal services is an important strategy people use to address the environmental pressures, especially the lack of services available in the neighbourhood and thus their use of transport services is a vital coping strategy (taxi or formally organised volunteer services);
- Strategies of using informal help. Neighbourhood cooperation for safety was an important strategy for increasing the feeling of safety, which was identified as an important environmental structural pressure. People actively developed good relationships with their neighbours and cultivated mutual help, as illustrated in the following statement: “We have a very good relationship with our neighbours such that when my son got married, no one was there until the late hours, the lady was 92 years old and she was ‘on guard duty’ until we got home.” (Focus group LJ, B6). Along with neighbours, friends offered some help, particularly to compensate for the lack of services and the distance from services. In line with the relevance of the family in Slovenia in care for the older people, the family cooperation and help was vital, ranging from repairs to adaptations of the dwellings, transport services, etc.;
- Strategies of moving oneself away from the environmental pressures. To address some of the mentioned environmental pressures, the respondents have moved away, i.e., changed their dwelling, or planned for such a move for the future: “In terms of maintenance, I have to say that previously I had to warm myself with wood, we also had oil, but I couldn’t do that anymore, so I went to live in a dwelling in an apartment block” (Focus Group Žalec, D1). Some informants also made occasional moves—retreats to places where there were fewer environmental pressures, such as retreating to a vacation home to escape the noise and density of the neighbourhood. Such occasional retreats were also seen on a smaller scale, for example, going for walks in nature to avoid the damp housing for a short time;
- Strategies of actively changing the environment. Housing renovation was an important strategy for dealing with environmental pressures, for instance, putting up safety rails in the bathroom, moving or discarding furniture to ensure greater space, moving things to lower shelves or adapting furniture to allow easier access. The informants were also very active in improving their neighbourhood. For example, they picked up litter on the streets in the neighbourhood to make it cleaner, and also addressed the authorities (in an attempt) to achieve more accessible services. “As for tidiness, you said tidiness of the living environment, I am cleaning our street at night. (…) I sweep it, I clean it every day, I take away the litter, every day” (Focus group LJ, B6).

#### 3.2.2. Attitudinal Adaptation 

Several strategies were identified in the category of attitudinal adaptation. Acceptance of and habituation to specific environmental pressures were viewed as important, as seen in the following quote:

“There’s an old scrap yard, in such a small place, I think, just like a landfill. That bothers me, but on the other hand, I’m working on the fact that… these are things you can’t improve, so that they don’t seem like, I don’t know what…, you have to adapt a little, and rather do something for yourself…” (Focus group Žalec, D2)

Another attitudinal adaptation was modesty and low expectations, which made the environmental pressures less important in the everyday life of an older person.

“I wash, I help myself with the wheelchair, then I walk a little with my cane, eat breakfast, take my medicine… my wife and I are both sick. She had cancer… (…) We talk about a lot of things. My wife and I have a good relationship, we can always talk about something. She also gardens. I also used to work a lot in the garden, when I was still able, we gardened together. (…) For me, my home and family are everything in the world. I feel good because I live with my wife and we get along well, and in my town I have all the basic things I need…” (Interviewee A2)

Distracting oneself from the environmental pressures was another important strategy where having contact with nature was especially relevant, also engaging in hobbies, etc., “as long as you can walk, and go out into nature, thank God, you go… There are those that close themselves off in the apartment, and complain and never go anywhere and have nothing to do” (Focus group Žalec, D8).

The final attitudinal strategy identified was planning for the future, which is an important aspect of attitudinal adaptation. Future planning typically included the contemplation of potential worse health and a greater need for care, and people actively reflected this with necessary moves to retirement homes, or moves to another apartment or by renovating their existing apartment.

“My husband and I have decided that as long as we help each other, we will stay that way, but if any problems arise, we will go to a secure apartment” (Focus group Žalec, D7).

## 4. Discussion

### 4.1. Environmental Pressures

We identified several structural environmental pressures within the housing accessibility and upkeep dimension. These problems have been proposed as being characteristic of central and eastern European countries where a large share of homeowners have difficulty with the upkeep of the dwelling and live in older buildings in urban areas [16]. One group of environmental pressures concerned safety issues and the renovations needed to make the space more suitable and safer in old age, particularly the bathroom and stairs needing a safety railing. The issue of unadapted and unsafe dwellings is noted in the literature and can cause an increase in falls and hospitalisations among older people [38,39,40], as well as make it difficult/impossible for the older people to engage in everyday activities [41,42,43]. Dangers in the living environment, problematic access and difficulties in performing day-to-day activities mean that an older person may experience a sense of helplessness, low self-esteem and reduced self-confidence. Individuals who have already fallen in their apartment may experience a strong sense of fear [44,45,46,47,48,49,50,51,52,53].

One important pressure identified in the study was formal environmental pressures linked to services. The availability and level of satisfaction with such services has been identified as a key factor in general satisfaction with the neighbourhood and an important reason for not wishing to move [54,55,56]. Urban areas generally have more accessible transportation and various services (shops, banks, etc.) than rural areas [57]. However, despite this, some areas can have worse access and smaller shops have frequently disappeared following the development of large shopping centres along the periphery [58,59]. The accessibility of services is a key aspect for enabling quality of ageing in place in Slovenia, as demonstrated by research in a comparative perspective [23]. Urban neighbourhoods often lack (at least some) vital services, where respondents pointed out transport services as being problematic. The strategies for addressing them, such as help from the family or formal transport services (also private) carry specific risks, either linked to the availability of such support from the family, or financial accessibility (e.g., of regular taxi journeys to needed services). The financial limitations are particularly relevant for the CEE region generally, and Slovenia in particular, due to higher poverty rates among older people than in the population in general [60].

Among the important informal environmental pressures identified, were the lack of family support and also poor relationships with neighbours. Neighbourly relations and socialising are core aspects of neighbourhood satisfaction, neighbourhood attachment and place identity [61,62]. Neighbourhood networks therefore represent an important aspect of social capital within the community that enable the neighbourhood to function well and address problems within it, emphasising the role of neighbourhood cohesion and social support and sense of belonging [62,63,64,65]. Therefore it is interesting that one of the coping strategies found was also the active nurturing of these relationships. These social aspects must be understood as an expression of human agency whereby older people function as actors in place-making [66]. The social dimension of the immediate environment therefore represents a resource and our findings indicate how this has been actively built by older people to address the environmental press, therefore making community-building an active strategy among older people. 

### 4.2. Coping Strategies and Capabilities

We identified several strategies within behavioural responses to environmental pressures, such as moving home to reduce environmental pressures and actively changing the environment, such as housing renovation. Furthermore, they improved entire neighbourhoods by actively cleaning them, for example. This is linked to what Buffel et al. [67] call collective actions aimed at promoting community safety and, in this case, general community well-being.

More passive coping strategies have been linked with acceptance and habituation as well as modesty and distraction. In our study, these are linked with resilience, therefore having a positive outlook on things, and a will to overcome the environmental stressors or not take them to heart. As was argued by [15], resilience is the developmental process of being mindful and prioritising behaviours, thoughts and feelings that facilitate contentment and can be understood as a phenomenon seen on the individual, contextual and larger sociocultural level. It is at once a process, an outcome and a resource, optimising opportunities for health and participation, and enhancing quality of life as people age, and is centrally linked with the coping process in stressful conditions (ibid.). 

This is also linked to understanding how an older person’s capabilities to develop these strategies are built up and supported or restrained. The capabilities approach puts forward the inequalities and constraints older people face in their choices [68]. Based on Sen’s work, capabilities are what an individual can potentially achieve, which is different from what people actually achieve—as the latter is linked to achieved functioning and individual agency [69]. Individual agency, if understood as the choices people make and their behaviours, can be conceptually linked to adaptive behaviours in P–E fit models. Capabilities are linked to individual-level conversion factors (the individual’s competencies, abilities, gender, class, ethnicity, etc.) as well as community conversion factors, i.e., how the environment enables individuals to convert their rights and available possibilities into real opportunities [69]. Capabilities therefore shift our attention from what people actually do to the possibilities they have for choice and distinguish between doing X and choosing to do X and actually doing it [70]. The behavioural and attitudinal coping strategies identified by our study must therefore be interpreted as linked to individual capabilities, which function as a conversion factor. The main capabilities include health and financial stability, which were linked and explicitly mentioned in the strategies of renovation, moving, future planning, use of services, etc. All of these strategies are heavily constrained by people’s financial resources. 

Our study identified no strategy that would indicate support from policy makers, municipal organisational structures or similar more formal support. Several of the strategies focused on community and family support networks. Yet, the need for additional support for answering these environmental pressures is vital (e.g., need for home modifications to improve safety and upkeep), as often the addressing of these pressures is beyond the individual’s capability and family’s support. This links with the considerable dependence on the family as support for older people, characteristic not only of Slovenia but also reflecting a familial character of the central and eastern European (CEE) region in care and the low mobility rates found in Slovenia [17]. A stronger focus should be given to policies for supporting, e.g., home modifications with formal policies and not only informal strategies, as we could observe. The problem of accessing good services and preventing bad experiences while engaging help with renovation is a vital policy direction, since older people in post-socialist cities tend to concentrate in older, lower quality housing that has a strong need for renovation [16]. 

### 4.3. Community Conversion Factors

In addition, community conversion factors are important, which we have also observed in our study, as part of environmental pressures. A well-functioning and close-knit neighbourhood facilitates the use of informal networks and neighbours as coping strategies. As has been discussed by various authors [62,71,72,73,74,75,76], public spaces and green spaces are important areas for meeting and linking bonds, as well as maintaining networks within a neighbourhood. The nearness of green areas is an important part of coping strategies, such as distracting oneself or moving away from environmental pressures (by taking walks in nature). Strengthening these capabilities consequently holds the potential to open up people’s strategies and choices and thus make independence and freedom the sum of all capabilities [68]. The community factors affect the coping strategies people have and the development of resilient attitudinal strategies. Strengthening community bonds is vital, as a sense of community is critical to resilient ageing [15]. Here the focus should not be on improving one-to one personal contact, but on ensuring structural solutions and addressing the structural factors that produce isolation and loneliness [65]. Therefore, individual, as well as neighbourhood factors, should also be seen as possible conversion factors that enable the use of particular coping strategies and the resulting specific person–environment fit. 

## 5. Conclusions

Understanding the person–environment fit and specific coping strategies formed in certain structural contexts can further assist in determining how the structural contexts (such as urban environments) can be changed to strengthen older people’s set of capabilities and improve their adaptive, coping strategies for dealing with the environmental pressures. Stressing the embeddedness of these strategies in local environments is important for future studies. It should be noted though, that we did not identify any clear strategies of decreasing risk, strategies of control or collective actions aimed at promoting community safety, as identified by Buffel et al. [67], which we associate with the specific context of the lower levels of urbanisation [77] and criminality in Slovenia’s [78] relatively small towns. This presents an important limitation of the study, as conditions and findings might differ significantly from those in much larger urban areas found in other central and eastern European countries.

We would also like to caution here, as underscored by Kelly and other researchers [12], that there is a tendency to overfocus on the micro aspects of everyday life while observing ageing in place and age-friendly neighbourhoods, causing broader aspects of social contexts to be overlooked. Resources to promote age-friendly urban neighbourhoods can be unequally distributed across neighbourhoods and within the older population [79]. Notwithstanding that this aspect is extremely important, the research data do not allow us to include this to any greater extent in the analysis and presents an additional limitation of this study which should be addressed in future research on the quality of ageing in urban environments. 

## Figures and Tables

**Table 1:** Environmental pressures in urban neighbourhoods in Slovenia.

Main Pressures	Specific Dimension	Examples
Structural/Housing	Accessibility	Stairs, no elevator, narrow doors and corridors steps between rooms, height of furniture
	Upkeep	Deteriorated appliances and furniture, insulation problems (dampness) and high heating costs, poor sound insulation, dwelling too big
	Safety	Fences, rails and other support in dwellings, unsuitable bathrooms—shower too small, slippery tiles
Structural/Neighbourhood	Walkability	Lack of benches to sit on, busy roads, hilly terrain in the vicinity
	Cleanliness	Poor maintenance of public spaces, noise pollution
	Safety	Vandalism and thefts, feeling unsafe, narrow and dangerous cycling lanes
	Lack of activities	Lack of space for activities for older-people, lack of space for associations to operate in
	Density	(Too) high density, lack of parking space
Informal Pressures	Family availability	Distance from family, lack of support from family
	Community functioning	Lack of a person with an ‘overview of things’, lack of opportunities for socialising, unfriendliness, poor relationships with neighbours
Formal Pressures	Accessibility of services	Lack of shops and services (banks, etc.), lack of cleaning facilities, accessibility of community health centres, lack of institutional care homes for older people, poor accessibility of social homecare
	Accessibility of transport services	Poor bus connections
	Quality of services	Lack of familiarity, impersonal services, lack of information

**Table 2:** Main coping strategies of older people in urban neighbourhoods in Slovenia.

Main Responses	Coping Strategies	Example
Behavioural Responses	Strategies of using formal help	Transportation services
	Strategies of using informal help and community building	Family cooperation Neighbourhood cooperation
	Strategies of moving oneself away from the environmental pressures	Moving to a vacation home
	Strategies of actively changing the environment	Housing modifications
Attitudinal Adaptation	Acceptance/habituation	Getting used to the noise from traffic in the neighbourhood
	Resilience	Not taking things ‘to heart’
	Keeping busy/using distractions	Going for walks in nature
	Modesty	Not demanding, e.g., additional shops
	Planning for the future	Move to homes for old people/a nursing home

## Data Availability

The data are in the process of submission to the publicly accessible Social Science Data Archives (Arhiv družboslovnih podatkov), Faculty of Social Sciences, University of Ljubljana. Available online: https://www.adp.fdv.uni-lj.si/eng/ (accessed on 1 March 2023).
